# Spousal concordance in adverse childhood experiences and the association with depressive symptoms in middle-aged and older adults: findings across China, the US, and Europe

**DOI:** 10.3389/fpubh.2023.1158590

**Published:** 2023-06-05

**Authors:** Weidi Sun, Ziyang Ren, Siyu Zhu, Siqing Cheng, Wen Liu, Ho Cheung William Li, Wei Xia, Changzheng Yuan, Davies Adeloye, Igor Rudan, Dexter Canoy, Peige Song

**Affiliations:** ^1^School of Public Health and Women’s Hospital, Zhejiang University School of Medicine, Hangzhou, Zhejiang, China; ^2^Institute of Reproductive and Child Health/Key Laboratory of Reproductive Health, National Health Commission of the People’s Republic of China, Peking University, Beijing, China; ^3^Department of Epidemiology and Biostatistics, School of Public Health, Peking University, Beijing, China; ^4^Department of Orthopedic Surgery, The Fourth Affiliated Hospital, International 16 Institutes of Medicine, Zhejiang University School of Medicine, Hangzhou, Zhejiang, China; ^5^Nethersole School of Nursing, The Chinese University of Hong Kong, Shatin, New Territories, Hong Kong, China; ^6^School of Nursing, Sun Yat-Sen University, Guangzhou, China; ^7^School of Public Health, Zhejiang University School of Medicine, Hangzhou, Zhejiang, China; ^8^Department of Nutrition, Harvard T. H. Chan School of Public Health, Boston, MA, United States; ^9^Centre for Global Health Research, Usher Institute of Population Health Sciences and Informatics, University of Edinburgh, Edinburgh, United Kingdom; ^10^Deep Medicine, Oxford Martin School, University of Oxford, Oxford, United Kingdom; ^11^Nuffield Department of Women’s and Reproductive Health, Medical Science Division, University of Oxford, Oxford, United Kingdom; ^12^NIHR Oxford Biomedical Research Centre, Oxford University Hospitals NHS Foundation Trust, Oxford, United Kingdom

**Keywords:** intra-familiar ACEs, extra-familiar ACEs, spousal concordance, depressive symptoms, middle-aged and older

## Abstract

**Background:**

Adverse childhood experiences (ACEs) are associated with higher depressive risks in adulthood. Whether respondents’ ACEs are associated with their own depressive symptoms in adulthood and whether this association extends to their spouses’ depressive symptoms remain unexplored.

**Methods:**

Data were from China Health and Retirement Longitudinal Study (CHARLS), the Health and Retirement Study (HRS), and the Survey of Health, Ageing and Retirement in Europe (SHARE). ACEs were categorized into overall, intra-familial, and extra-familial ACEs. Correlations of couples’ ACEs were calculated using Cramer’s V and partial Spearman’s correlation. Associations of respondents’ ACEs with spousal depressive symptoms were assessed using logistic regression, and mediation analyses were conducted to explore the mediating role of respondents’ depressive symptoms.

**Results:**

Significant associations between husbands’ ACEs and wives’ depressive symptoms, with odds ratios (ORs) and 95% confidence intervals (CIs) of 2.09 (1.36–3.22) for 4 or more ACEs in CHARLS, and 1.25 (1.06–1.48) and 1.38 (1.06–1.79) for 2 or more ACEs in HRS and SHARE. However, wives’ ACEs were associated with husbands’ depressive symptoms only in CHARLS and SHARE. Findings in intra-familial and extra-familial ACEs were consistent with our main results. Additionally, respondents’ depressive symptoms mediated more than 20% of the effect of respondents’ ACEs on spousal depressive symptoms.

**Conclusion:**

We found that ACEs were significantly correlated between couples. Respondents’ ACEs were associated with spousal depressive symptoms, with respondents’ depressive symptoms mediating the association. The bidirectional implications of ACEs on depressive symptoms should be considered within household and effective interventions are warranted.

## Introduction

Adverse childhood experiences (ACEs) encompass a wide spectrum of intra-familial and extra-familial traumatic events occurring in childhood, such as family violence and bullying (1, 2). The deleterious impacts of ACEs on life course health and related medical burdens have received increasing attention recently (3, 4). ACEs have been found to be significantly associated with increased risks of numerous diseases, particularly mental disorders like depression (5, 6).

Depression is a prevalent psychological disorder characterized by a persistent feeling of sadness and/or lack of pleasure (7). According to the Global Burden of Disease Study (GBD) 2019, there were 170.8 million cases of depression in 1990 and 279.6 million cases in 2019, corresponding to an increase of 63.7% (8). Depressive symptoms also significantly contribute to the development of chronic diseases and disabilities, imposing an enormous social and economic burden (9, 10). There is a growing understanding that adversities in childhood can heighten one’s risk of depression later in life. Some research also suggested considering numerical count and specific domains of ACEs on the association with depression (11).

Assortative mating theory posits the non-random pairing of similar individuals, leading to greater concordance in lifestyle or health conditions among couples (12). Recent research has revealed that newly married couples were consistent in their psychological conditions (12), suggesting that couples may share similar characteristics before marriage. However, it remains unclear whether couples have similar experiences in early childhood. Notably, depression appears to cluster within households, with previous literature identifying a correlation between depressive symptoms in older couples (13). Given the adverse implications of individuals’ ACEs on their mental health (13, 14), it is reasonable to deduce that ACEs in one spouse may induce his/her depressive symptoms and further extend to spousal depressive symptoms. However, there is limited research exploring this hypothesis.

To fill these research gaps and verify the above hypothesis across the social development spectrum, this study used population-based datasets from three regions with different social development circumstances: the China Health and Retirement Longitudinal Study (CHARLS) (15), the Health and Retirement Study (HRS) in the United States (the US) (16), and the Survey of Health, Ageing and Retirement in Europe (SHARE) (17) to investigate the spousal concordance of ACEs, the association between respondents’ ACEs and spousal depressive symptoms, and the mediating role of respondents’ depressive symptoms in this association.

## Materials and methods

### Data sources and study population

The CHARLS enrolled participants aged 45 years and above from 150 counties within 28 provinces across China, using a stratified multistage probability sampling strategy (15). The baseline survey was conducted in 2011, and participants were followed every 2 years. To date, three follow-up surveys have been conducted in 2013, 2015, and 2018. Information on childhood experiences was additionally collected in the 2014 life history survey from all living respondents in the 2011 and 2013 surveys.

The HRS is a population-based longitudinal survey of participants aged over 50 years in the United States. More details of the study design and recruitment can be found elsewhere (16). The initial cohort was established in 1992 based on a multi-stage area probability design, with several younger cohorts enrolled over time. Respondents were offered face-to-face or telephone follow-up interviews biennially from entry until 2018 (wave 14). The most updated and completed data on ACEs were collected in the 2012 survey.

The SHARE is a multidisciplinary and longitudinal study of people aged 50 years or older from 28 European countries and Israel (17). The survey was conducted biennially from 2004 until the present, with eight waves to date. In wave 3 in 2008 and wave 7 in 2017, the SHARELIFE retrospective interview was implemented to collect detailed information on participants’ life history including childhood experiences. Our study included participants from the 8th wave in 2018 (18, 19), and data on ACEs were from the 7th wave in 2017 (20, 21).

The CHARLS was approved by the Institutional Review Board at Peking University, the HRS was approved by the Institutional Reviewing Board at the University of Michigan and the National Institute on Aging, and the SHARE was approved by the Ethics Council of the Max Planck Society. Written informed consent was obtained from all respondents.

In this study, couples aged 50 years or older with complete data on ACEs and covariates were included. The study flow charts can be found in Supplementary Figures S1–S3.

### Definition of ACEs

Detailed definitions of ACEs are shown in Supplementary Table S1.

For the CHARLS, all ACEs before the age of 17 years were assessed by dichotomous or multiple-choice questions in the CHARLS 2014 life history questionnaire. We identified 14 ACE events, including 11 intra-familial domains (emotional neglect, family violence, parental separation or divorce, parental substance abuse, parents incarcerated, parental mental illness, parental disability, parental death, sibling death, physical abuse, and economic adversity) (22, 23) and 3 extra-familial domains (bullying, loneliness, and community violence) (24–26). All domains were further dichotomized and summed to obtain overall ACEs, intra-familial ACEs, and extra-familial ACEs, with values ranging from 0 to 14, 0 to 11, and 0 to 3, respectively. Overall ACEs were then classified as 0, 1, 2, 3, and 4 or more; intra-familial ACEs were classified as 0, 1, 2, 3, and 4 or more; and extra-familial ACEs were classified as 0, 1, and 2 or more.

In the HRS, the Psychosocial and Lifestyle Questionnaire was utilized to ask about childhood traumas before the age of 18 years (27). In this study, a total of 6 ACEs were identified, including 4 intra-familial domains (emotional neglect, parental substance abuse, physical abuse, and economic adversity) and 2 extra-familial domains (repeating school year and trouble with police) (28). Overall ACEs, intra-familial ACEs, and extra-familial ACEs were further categorized based on the number of ACEs, with values ranging from 0 to 6, 0 to 4, and 0 to 2, respectively. Overall ACEs were then classified as 0, 1, and 2 or more; intra-familial ACEs were classified as 0, 1, and 2 or more; and extra-familial ACEs were classified as 0, 1, and 2.

Lastly, the SHARE captured 6 ACEs, including 4 intra-familial domains (emotional neglect, absent biological parent, physical abuse, and economic adversity) and 2 extra-familial domains (non-parental abuse and loneliness) (29). All domains were summed to obtain overall ACEs, intra-familial ACEs, and extra-familial ACEs with values ranging from 0 to 6, 0 to 4, and 0 to 2, respectively. Overall ACEs were then classified as 0, 1, and 2 or more, intra-familial ACEs were classified as 0, 1, and 2 or more, and extra-familial ACEs were classified as 0, 1, and 2.

### Depressive symptoms assessment

In the CHARLS, depressive symptoms were assessed using the 10-item Center for Epidemiological Studies Depression Scale (CESD-10) (30). The validity of CESD-10 has been thoroughly demonstrated in the Chinese population (Cronbach *α* = 0.78) (31, 32). Participants were asked, “How you have felt and behaved during the last week,” with options including (1) rarely or none of the time (1 day), (2) some or a little of the time (1–2 days), (3) sometimes or a significant amount of the time (3–4 days), and (4) most or all of the time (5–7 days). This study assigned 0–3 to these four options, with a total depressive symptoms score ranging from 0 to 30. The cutoff score for depression was 10 (33, 34).

In the HRS, depressive symptoms were measured by the 8 item Center for Epidemiologic Studies Depression Scale (CESD-8), which has high specificity and sensitivity in identifying depressive cases (Cronbach *α* = 0.78) (35, 36). The CESD-8 contains six negative indicators and two positive indicators. The negative indicators asked whether the respondent experienced the following six emotions all or most of the time during the last week: (1) depressed, (2) everything is an effort, (3) sleep is restless, (4) felt alone, (5) felt sad, and (6) could not get going. The positive indicators asked whether the respondent felt happy or enjoyed life all or most of the time during the past week. The response “yes” to each negative indicator and the response “no” to each positive indicator obtained one point, with a total score for depressive symptoms ranging from 0 to 8. Scores ≥4 denoted having depressive symptoms (37, 38).

In the SHARE, depressive symptoms were assessed using the Europe-depression (EURO-D) scale (39, 40). This scale covers 12 sentiments of the respondent in the previous month, including depressed mood, pessimism, suicidal tendencies, guilt, sleep, interest, irritability, appetite, fatigue, concentration, enjoyment, and tearfulness. Each item takes 0 or 1 point, with the total score ranging from 0 to 12. Having depressive symptoms was identified with a score of 4 or above, which has proven to have great consistency and validity (Cronbach *α* = 0.61–0.80) (41, 42).

### Covariates

Information on age, sex, race (HRS only), residence (CHARLS and SHARE only), education, economic status, smoking history, and drinking history were self-reported through questionnaires at the baselines of CHARLS, HRS, and SHARE. Sex was divided into male and female in the three cohorts. In the HRS, race was categorized into White/Caucasian and Black or others (43, 44). Residence in the CHARLS and HRS was categorized into rural and urban. In the CHARLS, education was classified as primary school or less, middle school, and high school or above; in the HRS, education was classified as lower than high school, high school, college, and above college; and in the SHARE, education was measured by years spent in education. For economic status, we divided household annual or monthly income from each survey into bottom tertile, middle tertile, and top tertile. Smoking history was classified as never smoking and ever smoking in all three cohorts. Drinking history was classified as never drinking and ever drinking in the CHARLS and HRS; in the SHARE, drinking history was categorized into no recent drinking and recent drinking (45).

In the CHARLS, anthropometric data including body mass index (BMI), waist circumference (WC), and blood pressure were measured by healthcare workers. In the HRS, WC and blood pressure were measured by trained interviewers, while height and weight were self-reported. In the SHARE, only data on self-reported BMI were available. Hypertension was identified if one reported having physician-diagnosed hypertension, and/or taking anti-hypertensive drugs, and/or blood pressure ≥130/80 mmHg in the CHARLS and HRS. However, in the SHARE, only the first two criteria, physician-diagnosed hypertension and taking anti-hypertensive drugs, were used to define hypertension. In the three cohorts, diabetes, dyslipidemia (except for HRS), and cardiovascular diseases (CVDs) including stroke and heart diseases were defined by self-reported diagnoses, and/or taking related drugs.

### Statistical analysis

In the three cohorts, the baseline characteristics of included participants were described as medians with interquartile ranges (IQRs) for continuous variables, and frequencies and percentages (%) for categorical variables.

First, frequency tables were used to show the prevalence of spousal concordance of ACEs (46), with stratification of specific ACE domains. The correlations of each specific ACE between couples were calculated using the Chi-square test or Fisher’s exact test and this generated Cramer’s V. Furthermore, partial Spearman’s correlation test was used to calculate the correlations of overall ACEs, intra-familial ACEs, and extra-familial ACEs between couples. Model 1 was adjusted for both spouses’ age, race (HRS only), residence (CHARLS and SHARE only), education, and economic status. Model 2 was additionally adjusted for both spouses’ BMI, WC (CHARLS and HRS only), smoking history, and drinking history based on Model 1. Model 3 was further adjusted for both spouses’ diabetes, hypertension, dyslipidemia (CHARLS and SHARE only), and CVDs based on Model 2 (47–50).

After excluding couples with missing data on depressive symptoms in the three cohorts, logistic regression was used to investigate the association of respondents’ ACEs with spousal depressive symptoms. All ORs (95% CIs) were adjusted for spousal ACEs, age, race (HRS only), residence (CHARLS and SHARE only), education, economic status, BMI, WC (CHARLS and HRS only), smoking history, drinking history, diabetes, hypertension, dyslipidemia (CHARLS and SHARE only), and CVDs. We also assessed the association between respondents’ ACEs and depressive symptoms of their spouses who had never experienced ACEs. All the analyses mentioned above were conducted using SAS statistical software version 9.4 (SAS Institute).

**Figure 1:**
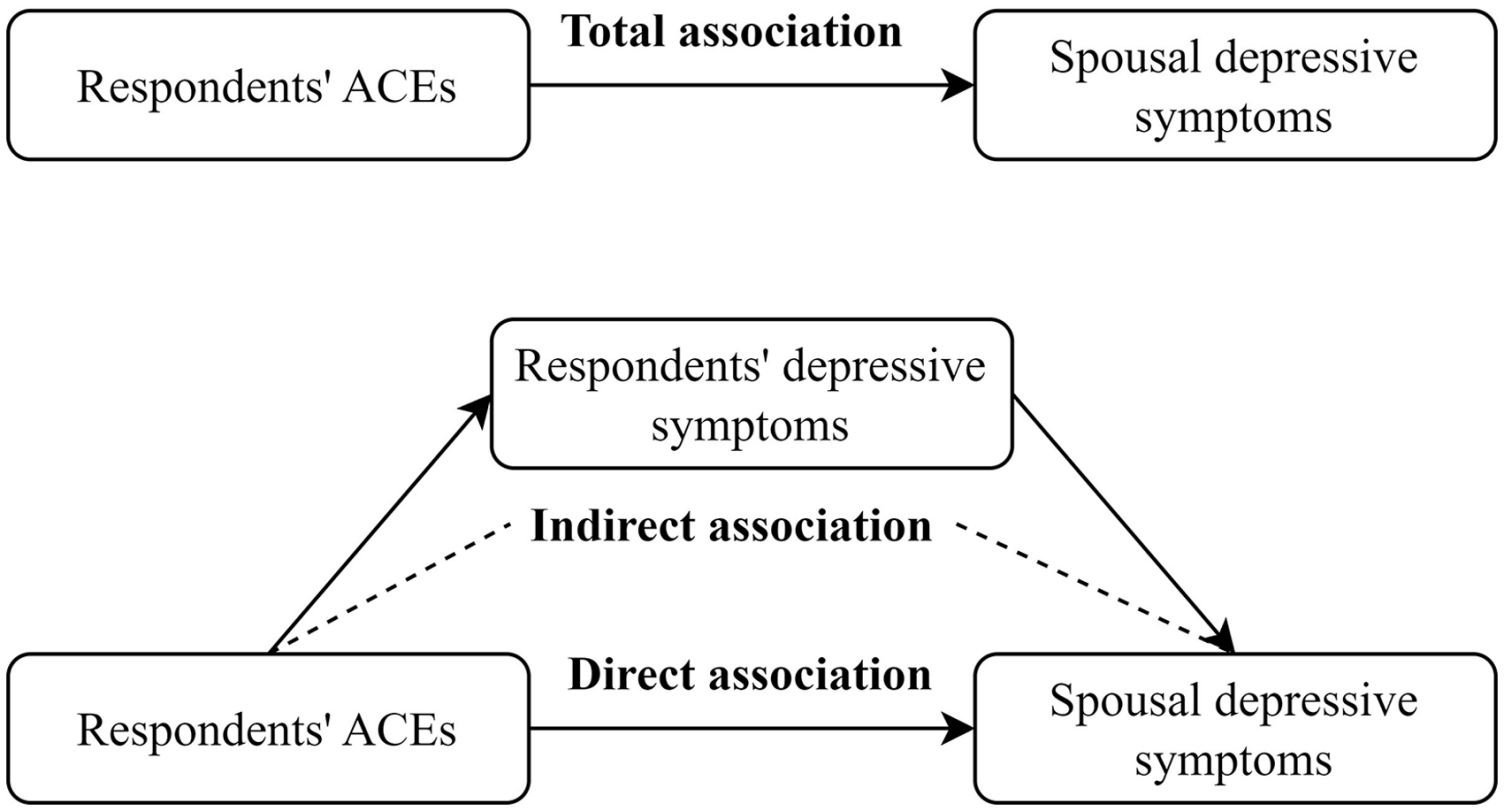
The mediation pathway of respondents’ depressive symptoms between respondents’ ACEs and spousal depressive symptoms. ACEs, adverse childhood experiences.

Finally, we performed mediation analyses using R statistical software version 4.1.2 (R Project for Statistical Computing) to explore whether respondents’ depressive symptoms mediated the effect of respondents’ ACEs on spousal depressive symptoms (Figure 1).

Reporting of this study was done under Strengthening the Reporting of Observational studies in Epidemiology (STROBE) guidelines (51). All analyses were two-sided, and a *p* value of <0.05, a 95% confidence interval (CI) of odds ratio (OR) that did not cross 1.00, or a 95% CI of Spearman’s correlation or *β* that did not cross 0 was considered statistically significant.

## Results

### Participants’ characteristics

The baseline characteristics of participant couples from the CHARLS, HRS, and SHARE are shown in Supplementary Table S2. Among 2,394 couples in the CHARLS, more than 90% of these couples experienced ACEs. More specifically, intra-familial ACEs affected 92.2% of husbands and 89.1% of wives, while 29.5% of husbands and 27.2% of wives reported having experienced extra-familial ACEs. Among 3,131 couples from the HRS, 60.4% of husbands and 52.2% of wives reported having ACEs. The proportions of husbands exposed to intra-familial ACEs and extra-familial ACEs were 48.4 and 29.0%, while of wives were 48.4 and 11.8%. Among 1,831 spouses in the SHARE, about 60% suffered from ACEs. Intra-familial ACEs affected 53.8% of husbands and 50% of wives, while extra-familial ACEs impacted 29.7% of husbands and 23.1% of wives.

### Spousal concordance of ACEs

The majority (more than 50%) of couples from the three cohorts were in concordance across specific and multiple domains of ACEs (Table 1). In the CHARLS, domain-specific analyses indicated significant correlations between couples except for parental separation or divorce, parents incarcerated, and sibling death. In the HRS, significant correlations of all specific ACEs were found between couples. Among those in the SHARE, noticeable correlations were found except for absent biological parents. After adjusting for both couples’ characteristics, overall ACEs, intra-familial ACEs, and extra-familial ACEs showed substantial correlations between spouses in the CHARLS, HRS, and SHARE (Supplementary Table S3).

**Table 1:** Spousal concordance of domains of ACEs in the CHARLS, HRS, and SHARE.

Domains of ACEs	Concordant (%)		Discordant: 1 spouse had (%)	Cramer’s V	*p* value
	Both had	Both did not have			
*The CHARLS*					
*Specific domains*					
Emotional neglect	19.0	39.8	41.2	0.14	<0.001
Family violence	7.9	60.1	32.1	0.12	<0.001
Parental separation or divorce	0.0	99.0	1.0	/	1.000
Parental substance abuse	37.2	25.1	37.7	0.24	<0.001
Parents incarcerated	0.0	99.5	0.5	/	1.000
Parental mental illness	2.9	80.2	16.9	0.16	<0.001
Parental disability	5.9	64.1	30.0	0.09	<0.001
Parental death	2.2	76.2	21.6	0.05	0.029
Sibling death	0.3	90.6	9.1	0.02	0.489
Physical abuse	11.9	51.9	36.2	0.15	<0.001
Economic adversity	16.4	44.6	39.1	0.15	<0.001
Bullying	4.3	71.1	24.6	0.12	<0.001
Loneliness	2.0	81.9	16.1	0.11	<0.001
Community violence	2.4	82.2	15.5	0.15	<0.001
*Overall ACEs*				0.20	<0.001
1 or more	85.5	1.4	13.1		
2 or more	54.9	10.2	34.9		
3 or more	28.0	28.5	43.5		
4 or more	11.5	51.7	36.8		
*Intra-familial ACEs*				0.17	<0.001
1 or more	83.2	2.0	14.8		
2 or more	49.4	13.2	37.5		
3 or more	20.2	35.2	44.6		
4 or more	6.4	62.5	31.2		
*Extra-familial ACEs*				0.17	<0.001
1 or more	11.5	54.8	33.7		
2 or more	0.8	86.9	12.3		
*The HRS*					
*Specific domains*					
Emotional neglect	3.3	70.7	26.0	0.05	0.010
Parental substance abuse	4.5	68.1	27.4	0.08	<0.001
Physical abuse	0.9	86.2	12.8	0.06	0.002
Economic adversity	10.2	53.7	36.1	0.11	<0.001
Repeating school year	3.6	72.6	23.8	0.12	<0.001
Trouble with police	0.6	86.4	13.0	0.09	<0.001
*Overall ACEs*				0.14	<0.001
1 or more	34.3	21.7	44.0		
2 or more	7.7	59.4	32.9		
*Intra-familial ACEs*				0.10	<0.001
1 or more	25.8	29.0	45.2		
2 or more	3.7	71.3	25.0		
*Extra-familial ACEs*				0.10	<0.001
1 or more	4.8	63.9	31.3		
2	0.2	95.8	4.1		
*The SHARE*					
*Specific domains*					
Emotional neglect	8.1	63.9	28.0	0.19	<0.001
Absent biological parent	1.9	78.4	19.8	0.05	0.055
Physical abuse	7.7	66.9	25.5	0.23	<0.001
Economic adversity	11.6	63.8	24.6	0.33	<0.001
Non-parental abuse	1.0	91.3	7.7	0.17	<0.001
Loneliness	7.4	70.5	22.1	0.27	<0.001
*Overall ACEs*				0.29	<0.001
1 or more	41.8	22.4	35.8		
2 or more	11.3	56.7	32.0		
*Intra-familial ACEs*				0.24	<0.001
1 or more	32.3	28.5	39.3		
2 or more	6.7	66.6	26.8		
*Extra-familial ACEs*				0.27	<0.001
1 or more	8.9	66.1	25.0		
2	0.2	96.3	3.6		

ACEs, adverse childhood experiences; CHARLS, China Health and Retirement Longitudinal Study; HRS, Health and Retirement Study; SHARE, Survey of Health, Ageing and Retirement in Europe.

### Association of respondents’ ACEs with spousal depressive symptoms

Overall, 2,263 husbands (24.2% cases of depressive symptoms) and 2,203 wives (38.0% cases of depressive symptoms) were included in the CHARLS. We found significant associations between husbands’ ACEs and wives’ depressive symptoms, with fully adjusted ORs (95% CI) of 1.61 (1.03 to 2.50), 2.09 (1.36 to 3.22), and 1.13 (1.07 to 1.19) for 2, 4 or more, and continuous ACEs, respectively. On the other hand, husbands married to wives with ACEs (continuous) had a significantly higher risk of depressive symptoms (OR = 1.07, 95% CI 1.02 to 1.13), as shown in Table 2. Supplementary Table S4 also shows that respondents’ intra-familial and extra-familial ACEs had substantial associations with spousal depressive symptoms.

**Table 2:** Association between respondents’ overall ACEs and spousal depressive symptoms in the CHARLS, HRS, and SHARE.

	Husbands’ ACEs on wives’ depressive symptoms	Wives’ ACEs on husbands’ depressive symptoms
	OR (95% CI)	
*The CHARLS*		
No. of participants	2,203	2,263
0	Reference	Reference
1	1.41 (0.89 to 2.23)	1.06 (0.70 to 1.60)
2	1.61 (1.03 to 2.50)	0.91 (0.60 to 1.38)
3	1.46 (0.93 to 2.29)	1.11 (0.74 to 1.69)
4 or more	2.09 (1.36 to 3.22)	1.44 (0.98 to 2.14)
Continuous	1.13 (1.07 to 1.19)	1.07 (1.02 to 1.13)
*The HRS*		
No. of participants	3,119	3,099
0	Reference	Reference
1	1.18 (1.01 to 1.39)	1.14 (0.97 to 1.34)
2 or more	1.25 (1.06 to 1.48)	1.07 (0.89 to 1.28)
Continuous	1.08 (1.02 to 1.15)	1.02 (0.96 to 1.10)
*The SHARE*		
No. of participants	1805	1786
0	Reference	Reference
1	1.11 (0.86 to 1.43)	1.03 (0.77 to 1.39)
2 or more	1.38 (1.06 to 1.79)	1.80 (1.33 to 2.44)
Continuous	1.16 (1.05 to 1.27)	1.24 (1.11 to 1.39)

ACEs, adverse childhood experiences; CHARLS, China Health and Retirement Longitudinal Study; HRS, Health and Retirement Study; SHARE, Survey of Health, Ageing and Retirement in Europe; OR, odds ratio; CI, confidence interval. All ORs (95% CIs) between respondents’ (husbands’ or wives’) ACEs and spousal (wives’ or husbands’) depressive symptoms were adjusted for spousal ACEs, age, race (HRS only), residence (CHARLS and SHARE only), education, economic status, body mass index, waist circumference (CHARLS and HRS only), smoking history, drinking history, diabetes, hypertension, dyslipidemia (CHARLS and SHARE only), and cardiovascular diseases.

In the HRS, a total of 3,099 husbands and 3,119 wives were included, of whom around 10% had depressive symptoms. Wives whose husbands had 2 or more ACEs were more likely to have depressive symptoms (OR = 1.25, 95% CI 1.06 to 1.48) compared to those whose spouses had no ACE. However, wives’ ACEs were not significantly associated with husbands’ depressive symptoms. Associations of respondents’ intra-familial and extra-familial ACEs with spousal depressive symptoms were in line with the main findings (Supplementary Table S4).

In the SHARE, the proportion of depressive symptoms among the included 1,786 husbands and 1,850 wives were 21.4 and 32.7%, respectively. It was found that wives married to husbands with 2 or more ACEs (vs. 0 ACE) were at a higher risk of having depressive symptoms, with fully adjusted ORs (95% CIs) of 1.38 (1.06 to 1.79). Similarly, wives’ ACEs were associated with husbands’ depressive symptoms, with ORs (95% CIs) of 1.80 (1.33 to 2.44) for 2 or more ACEs as shown in Table 2. Significant associations were also found between 2 or more respondents’ intra-familial ACEs and spousal depressive symptoms, as well as wives’ extra-familial ACEs and husbands’ depressive symptoms (Supplementary Table S4).

Furthermore, the associations between respondents’ ACEs and the depressive symptoms of spouses who had no ACEs are shown in Table 3. In the CHARLS, we did not observe significant associations between respondents’ ACEs and spousal depressive symptoms. In the HRS, the ORs (95% CIs) of depressive symptoms of wives married to husbands with 1 ACE and 2 or more ACEs (vs. 0 ACE) were 1.32 (1.05 to 1.66) and 1.40 (1.08 to 1.81), respectively. Wives’ 1 ACE was also found to be associated with husbands’ depressive symptoms. In the SHARE, a substantial association was only found with depressive symptoms of husbands whose wives had 2 or more ACEs (vs. 0 ACE) (OR = 2.04, 95% CI 1.15 to 3.61).

**Table 3:** Association between respondents’ ACEs and the depressive symptoms of spouses who had no ACEs in the CHARLS, HRS, and SHARE.

	Husbands’ ACEs on wives’ depressive symptoms	Wives’ ACEs on husbands’ depressive symptoms
	OR (95% CI)	
*The CHARLS*		
No. of participants	211	152
0	Reference	Reference
1	1.47 (0.47 to 4.58)	2.79 (0.62 to 12.50)
2 or more	1.22 (0.45 to 3.33)	1.15 (0.27 to 4.99)
Continuous	1.06 (0.88 to 1.27)	0.84 (0.60 to 1.17)
*The HRS*		
No. of participants	1,495	1,239
0	Reference	Reference
1	1.32 (1.05 to 1.66)	1.45 (1.11 to 1.89)
2 or more	1.40 (1.08 to 1.81)	1.23 (0.89 to 1.71)
Continuous	1.14 (1.04 to 1.26)	1.12 (0.99 to 1.27)
*The SHARE*		
No. of participants	733	710
0	Reference	Reference
1	0.93 (0.61 to 1.42)	0.93 (0.56 to 1.55)
2 or more	1.14 (0.72 to 1.81)	2.04 (1.15 to 3.61)
Continuous	1.05 (0.88 to 1.26)	1.23 (0.99 to 1.55)

ACEs, adverse childhood experiences; CHARLS, China Health and Retirement Longitudinal Study; HRS, Health and Retirement Study; SHARE, Survey of Health, Ageing and Retirement in Europe; OR, odds ratio; CI, confidence interval. All ORs (95% CIs) between respondents’ (husbands’ or wives’) ACEs and spousal (wives’ or husbands’) depressive symptoms were adjusted for spousal age, race (HRS only), residence (CHARLS and SHARE only), education, economic status, body mass index, waist circumference (CHARLS and HRS only), smoking history, drinking history, diabetes, hypertension, dyslipidemia (CHARLS and SHARE only), and cardiovascular diseases.

### Mediation of respondents’ ACEs and spousal depressive symptoms by respondents’ depressive symptoms

Finally, we found that respondents’ depressive symptoms mediated the association between respondents’ ACEs and spousal depressive symptoms, as shown in Table 4, which indicated that respondents with ACEs had worse depressive symptoms which in turn contributed to worse depressive symptoms in their spouses. More specifically, husbands’ depressive symptoms accounted for 23.5% (5.2 to 52.0%), 24.2% (2.7 to 129.5%), and 29.4% (4.5 to 79.2%) of the association between husbands’ ACEs and wives’ depressive symptoms in the CHARLS, HRS, and SHARE, respectively. Furthermore, wives’ depressive symptoms mediated 67.9% (23.1 to 270.9%) and 36.9% (14.7 to 71.7%) of the effect of wives’ ACEs on husbands’ depressive symptoms in the CHARLS and SHARE, respectively.

**Table 4:** Adjusted direct and indirect associations of respondents’ ACEs with spousal depressive symptoms via respondents’ depressive symptoms.

	Husbands’ ACEs		Wives’ ACEs	
	*β* (95% CI)	*p* value	*β* (95% CI)	*p* value
*CHARLS*				
No. of participants	2,110	NA	2,110	NA
Total association	0.022 (0.011 to 0.032)	<0.001	0.010 (0.002 to 0.018)	<0.001
Direct association	0.017 (0.006 to 0.026)	<0.001	0.003 (−0.005 to 0.011)	0.540
Indirect association via spousal depressive symptoms	0.005 (0.001 to 0.010)	0.008	0.007 (0.002 to 0.012)	<0.001
Proportion mediated, %	23.5 (5.2 to 52.0)	0.008	67.9 (23.1 to 270.9)	<0.001
*HRS*				
No. of participants	3,088	NA	3,088	NA
Total association	0.009 (0.001 to 0.017)	0.034	0.003 (−0.006 to 0.011)	0.450
Direct association	0.007 (−0.002 to 0.016)	0.110	0.002 (−0.007 to 0.010)	0.626
Indirect association via spousal depressive symptoms	0.002 (0.001 to 0.003)	0.004	0.001 (0 to 0.003)	0.142
Proportion mediated, %	24.2 (2.7 to 129.5)	0.038	19.8 (−292.2 to 321.9)	0.168
*SHARE*				
No. of participants	1766	NA	1766	NA
Total association	0.030 (0.009 to 0.050)	0.002	0.031 (0.014 to 0.047)	<0.001
Direct association	0.021 (0.002 to 0.039)	0.024	0.020 (0.004 to 0.033)	0.010
Indirect association via spousal depressive symptoms	0.009 (0.001 to 0.018)	0.026	0.012 (0.004 to 0.019)	0.002
Proportion mediated, %	29.4 (4.5 to 79.2)	0.028	36.9 (14.7 to 71.7)	0.002

ACEs, adverse childhood experiences; CI, confidence interval; CHARLS, China Health and Retirement Longitudinal Study; HRS, Health and Retirement Study; SHARE, Survey of Health, Ageing and Retirement in Europe. All models were adjusted for spousal ACEs, age, race (HRS only), residence (CHARLS and SHARE only), education, economic status, smoking history, drinking history, body mass index, waist circumference (CHARLS and HRS only), diabetes, hypertension, dyslipidemia (CHARLS and SHARE only), and cardiovascular diseases.

## Discussion

Across the CHARLS, HRS, and SHARE, there was a considerable concordance of overall ACEs, intra-familial ACEs, and extra-familial ACEs between couples. Notably, respondents’ ACEs, particularly the intra-familial ACEs, were significantly linked to an elevated risk of spousal depressive symptoms across all three cohorts, with more than 20% of these associations being attributed to respondents’ own depressive symptoms. The only exception was wives’ ACEs and husbands’ depressive symptoms in the HRS. These results provide valuable insights into the transmission of the effects of ACEs within a household and highlight the importance of adopting a life-course and family-based perspective in mental health interventions.

This study added unique findings about the concordance in ACEs among couples, which can be partly explained by the assortative mating theory, proposing that people are inclined to marry partners who are similar to them in various aspects, such as socioeconomic status, lifestyle, and even cardiovascular risk (46, 52, 53). A recent study also reported that women who experienced ACEs tended to have husbands with ACEs as well (54). Moreover, ACEs themselves can increase the risks of lower socioeconomic status, substance abuse, and cardiovascular risks, then those consequences may further influence mate choice in different ways (4, 55). On the individual level, ACEs have been found to increase risks of various later-life diseases (4). Therefore, it is plausible that couples both having ACEs might be more vulnerable to negative health outcomes due to the synergistic effects. Further research on the underlying mechanisms behind this phenomenon is warranted.

We also found significant associations between respondents’ ACEs and spousal depressive symptoms in middle-aged and older adults even after adjusting for spousal ACEs. These associations were found to be mediated by respondents’ depressive symptoms, highlighting the extensive impacts of ACEs on individuals and households. Our findings emphasized the importance of implementing specific interventions at the couple-level or household-level. It is widely recognized that individuals with ACEs are prone to developing depression (1, 56). Previous studies have revealed that spouses of those who suffer from depression experience high levels of marital distress and low levels of marital satisfaction, which may further exacerbate their depressive symptoms (57, 58). In addition, respondents’ depressive symptoms can independently aggravate spousal depressive symptoms (59). Physically, ACEs can exacerbate the hypothalamic–pituitary–adrenal (HPA) axis dysfunction by inducing couple conflict, resulting in reduced abilities of vital organs to respond to environmental stimulation like stress and emotion, leading to depressive symptoms in spouses (60–63). From a couple-based relationship perspective, the stress of one person has marked impacts on other intimate members in the household, which is called stress contagion within couples (14, 64). Relationship distress often occurs because couples frequently interact in maladaptive ways around depressive symptoms, whether one or both partners suffer from them. Due to the interdependence of spousal lives, the presence of relationship distress can predict the development of depressive symptoms in one partner, and this increases the possibility of the other spouse becoming distressed and obtaining depressive symptoms (65).

Interestingly, this study found that the association of husbands’ ACEs with wives’ depressive symptoms was more significant than vice versa. Prior research has shown that wives respond more than husbands to their spouses’ chronic health conditions, and this aligns with the results from this study (66). Furthermore, when couples experienced negative communications, wives tended to have worse sleep quality than husbands (67). Poorer sleep can further stimulate the production of pro-inflammatory cytokines and promote depressive symptoms (68). Given that women are more affected by sleep disorders and inflammation than men (69), ACEs may induce increased marital conflict and render wives more susceptible to depressive symptoms.

To the best of our knowledge, this is the first and most comprehensive study to explore the spousal concordance of ACEs, the association between respondents’ ACEs and spousal depressive symptoms, and the mediating role of respondents’ depressive symptoms in this association. We used data from three rigorous and valid surveys, the CHARLS, HRS, and SHARE from China, the United States, and Europe, respectively. These three regions represent three different levels of development in terms of public welfare: China is a developing country, the US is a developed country with a high disparity, and Europe is well-developed with good public welfare. The results of the study showed that across these populations, ACEs are significantly correlated between couples, and respondents’ depressive symptoms mediated more than 20% of the effect of respondents’ spousal ACEs on depressive symptoms. In addition, this study divided overall ACEs into intra-familial and extra-familial ACEs to explore their spousal concordance and associations with spousal depression, filling an important gap in the literature. However, there are some limitations in our research. First, information on ACEs was collected via self-reported questionnaires, which may have led to recall bias. Second, the specific items of ACEs and definitions of several covariates differ across the three cohorts because of different questionnaire designs. Finally, due to the retrospective design, the sequence in which couples experience depressive symptoms is unclear.

## Conclusion

We found that overall, intra-familial, and extra-familial ACEs were significantly correlated among couples. Additionally, respondents’ ACEs were associated with spousal depressive symptoms, with respondents’ depressive symptoms mediating the correlation. This highlights the need to consider the implications of childhood adversity on later-life depressive symptoms at the couple-level or within household. It is necessary to strengthen effective interventions for ACEs and depressive symptoms to reduce the detrimental influence on individuals and the two-way influence between couples.

## Data availability statement

Publicly available datasets were analyzed in this study. The data that support the findings of this study are available from the websites of China Health and Retirement Longitudinal Study at http://charls.pku.edu.cn/en, Health and Retirement Study at https://hrsdata.isr.umich.edu/, and Survey of Health, Ageing and Retirement in Europe at https://share-eric.eu.

## Author contributions

PS and ZR designed the study. ZR managed and analyzed the data. ZR and WS prepared the first draft. ZR, WS, SZ, SC, and WL reviewed and edited the manuscript, with comments from PS, HL, WX, CY, DA, IR, and DC. All authors contributed to the article and approved the submitted version.

## Acknowledgments

We are grateful to the China Center for Economic Research at Beijing University for providing us with the data, and we thank the CHARLS research and field team for collecting the data. The HRS (Health and Retirement Study) is sponsored by the National Institute on Aging (grant number NIA U01AG009740) and is conducted by the University of Michigan. The SHARE data collection has been funded by the European Commission, DG RTD through FP5 (QLK6-CT-2001-00360), FP6 (SHARE-I3: RII-CT-2006-062193, COMPARE: CIT5-CT-2005-028857, SHARELIFE: CIT4-CT-2006-028812), FP7 (SHARE-PREP: GA no. 211909, SHARE-LEAP: GA no. 227822, SHARE M4: GA no. 261982, DASISH: GA no. 283646) and Horizon 2020 (SHARE-DEV3: GA no. 676536, SHARE-COHESION: GA no. 870628, SERISS: GA no. 654221, SSHOC: GA no. 823782, SHARE-COVID19: GA no. 101015924) and by DG Employment, Social Affairs & Inclusion through *VS* 2015/0195, *VS* 2016/0135, *VS* 2018/0285, *VS* 2019/0332, and *VS* 2020/0313. Additional funding from the German Ministry of Education and Research, the Max Planck Society for the Advancement of Science, the U.S. National Institute on Aging (U01_AG09740-13S2, P01_AG005842, P01_AG08291, P30_AG12815, R21_AG025169, Y1-AG-4553-01, IAG_BSR06-11, OGHA_04-064, HHSN271201300071C, and RAG052527A), and from various national funding sources is gratefully acknowledged (see www.share-project.org).

## Conflict of interest

The authors declare that the research was conducted in the absence of any commercial or financial relationships that could be construed as a potential conflict of interest.

## Publisher’s note

All claims expressed in this article are solely those of the authors and do not necessarily represent those of their affiliated organizations, or those of the publisher, the editors and the reviewers. Any product that may be evaluated in this article, or claim that may be made by its manufacturer, is not guaranteed or endorsed by the publisher.

## Supplementary material

The Supplementary material for this article can be found online at: https://www.frontiersin.org/articles/10.3389/fpubh.2023.1158590/full#supplementary-material
